# Intranasal Application of *S. epidermidis* Prevents Colonization by Methicillin-Resistant *Staphylococcus aureus* in Mice

**DOI:** 10.1371/journal.pone.0025880

**Published:** 2011-10-05

**Authors:** Bonggoo Park, Tadayuki Iwase, George Y. Liu

**Affiliations:** 1 Division of Infectious Diseases, Department of Pediatrics, Research Division of Immunology, Department of Biomedical Sciences, and the Immunobiology Research Institute, Cedars-Sinai Medical Center, Los Angeles, California, United States of America; 2 Department of Bacteriology, The Jikei University School of Medicine, Tokyo, Japan; Columbia University, United States of America

## Abstract

Methicillin-resistant *S. aureus* emerged in recent decades to become a leading cause of infection worldwide. Colonization with MRSA predisposes to infection and facilitates transmission of the pathogen; however, available regimens are ineffective at preventing MRSA colonization. Studies of human nasal flora suggest that resident bacteria play a critical role in limiting *S. aureus* growth, and prompted us to query whether application of commensal resident bacteria could prevent nasal colonization with MRSA. We established a murine model system to study this question, and showed that mice nasally pre-colonized with *S. epidermidis* became more resistant to colonization with MRSA. Our study suggests that application of commensal bacteria with antibiotics could represent a more effective strategy to prevent MRSA colonization.

## Introduction

Methicillin-resistant *Staphylococcus aureus* (MRSA) colonization poses a major public health problem because it predisposes colonized individuals to infection and facilitates spread of the pathogen to close contacts [1]. Attempts to address this problem have led to the widespread practice of MRSA decolonization both in healthcare-settings and in the community [1]. However, as a number of studies have suggested, standard regimens prescribed by most physicians do not prevent colonization of patients [2]. For example, individuals who receive a routine course of mupirocin in the nares and a body wash with hexachlorophene are frequently found to be colonized within few months, especially in the setting of close contact with MRSA colonizers [2]. While extended application of nasal mupirocin can be more effective, it leads to unacceptable level of mupirocin resistance [1], [3]. Based on studies of available regimens, currently there is no effective solution to prevent MRSA colonization.

An approach that showed promise in the 1960's was a strategy called bacterial interference [4], in which a less virulent *S. aureus* strain was used to block colonization by pathogenic *S. aureus* strains. Application of this strategy in the setting of *S. aureus* outbreaks proved to be effective, as patients treated with the “nonpathogenic“ 502A *S. aureus* strain showed a significant decrease in infection rate in multiple trials [4]. Unfortunately 502A was eventually linked to cases of *S. aureus* infections, and therefore enthusiasm for this strategy was dampened [5].

Notwithstanding the results of these investigations, many studies since have shown that select resident bacteria actively compete against *S. aureus* for survival on human skin and mucosal surfaces [6], [7], [8]. For example, following introduction of the seven-valent pneumococcal vaccine, researchers noted increased *S. aureus* colonization and infection among vaccinees, suggesting that removal of *S. pneumoniae* from the human nose permitted *S. aureus* to colonize more freely [6], [9]. We recently showed that *S. aureus* elaboration of catalase protected the pathogen from killing by *S. pneumoniae* [10]. However, even with the expression of catalase, *S. aureus* resistance to *S. pneumoniae* killing is limited [10], [11].

Human studies have also confirmed that nasal commensal bacteria such as *S. epidermidis* and *Corynebacterium* spp. compete with *S. aureus* for the same niche, as presence of one often predicts absence of the other in the same individual [8], [12], [13]. In our study, we showed that intranasal application of *S. epidermidis*, which secretes Esp [13], prevented *S. aureus* nasal colonization. These findings prompted us to ask whether the current regimen, consisting of the use of topical antibiotics, could be improved by co-administration of a competing commensal bacterium to prevent MRSA colonization.

## Results

### Establishment of the murine experimental model

**Figure 1:**
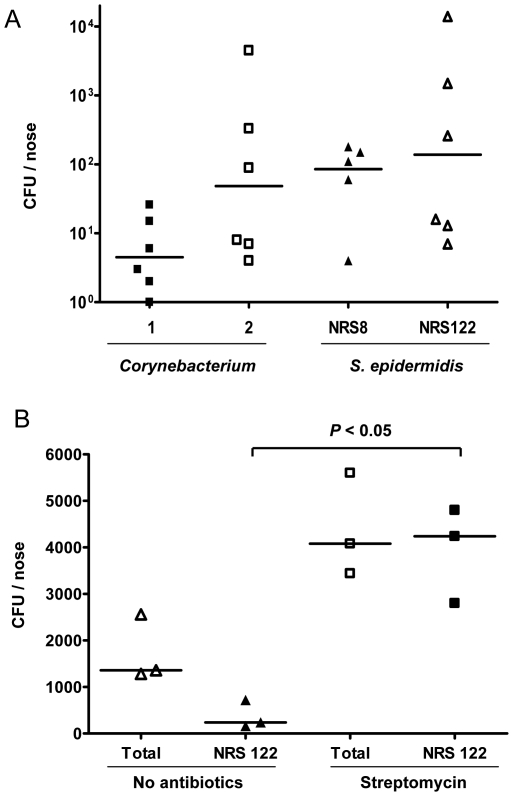
Optimizing conditions for bacterial interference. (A) Efficacy of nasal colonization by four strains of bacteria. CD1 mice were inoculated with 1×10^9^ CFU on d1 and 4. Nasal CFU was enumerated 3 days after the last inoculation. *Corynebacterium* spp. 1 versus NRS122: *p*<0.05. (B) Effect of oral streptomycin treatment on *S. epidermidis* NRS122 nasal colonization. Mice were given streptomycin water for 7 days, then inoculated with a streptomycin-resistant strain of NRS122 daily for 3 days. Nasal CFU were harvested 3 days later. Shown are total CFU or CFU from streptomycin plates. Bars in the graph represent the median CFU of each experimental group.

To determine whether application of commensal bacteria could prevent nasal colonization of mice with MRSA, we first tested a number of candidate strains for intranasal survival. We reasoned that bacteria that could compete successfully against MRSA must first colonize the nose at high concentration. Prior studies have suggested that both *Corynebacterium* spp. and *S. epidermidis* interfere with *S. aureus* colonization in the nose of human subjects [8], [12], [13]. In Figure 1A, we inoculated the nose of CD1 mice with 2 strains of *Corynebacterium* spp. and 2 strains of *S. epidermidis*, and sacrificed the mice after 3 days to enumerate surviving nasal CFU. Based on this experiment, *S. epidermidis* NRS122 colonized the nares of mice as well or better than the other strains, and was therefore selected as our candidate strain.

In preliminary experiments, we noted that both MRSA and *S. epidermidis* colonize the nose of 8–12 week old mice poorly. To fine-tune our murine model and optimize nasal *S. epidermidis* and MRSA colonization, we next investigated the impact of age of the host. In a prior study, our lab has demonstrated that 6 month old mice show a more limited immune response and carry a higher *S. aureus* burden compared to 8–12 week old mice following a subcutaneous infection [14]. Therefore we speculated that it would be possible to increase *S. epidermidis* (and MRSA) colonization using 6 month old mice. We applied *S. epidermidis* NRS122 to the nares of 6–12 week old mice and 6 month old mice, and showed that the nares of 6 month old mice were more prominently colonized with NRS122 compared to 6–12 week old mice after 3 days (5560 versus 770 CFU: *p*<0.05; n = 4–5 per group).

Next we sought to simulate the clinical scenario in which patients received an antibiotic as part of the decolonizing regimen, and to investigate whether application of *S. epidermidis* protected from MRSA colonization. The use of topical mupirocin (used routinely in human) is impractical in mice because of the narrow opening of nares which prevented consistent application of the thick antibiotic gel. Therefore, we elected instead to use oral streptomycin, an antibiotic that effectively cleared the endogenous nasal flora in mice [15]. To verify that application of streptomycin does not lead to inadvertent killing of the competing *S. epidermidis*, we streaked NRS122 on streptomycin agar plates (THA with 500 µg/ml streptomycin), and isolated colonies of NRS122 that are resistant to streptomycin. Next, we treated mice orally with normal drinking water or water supplemented with 1000 µg/ml streptomycin for 7 days. On day 3 after the initiation of streptomycin, we inoculated both groups of mice with the streptomycin-resistant *S. epidermidis* NRS122 once a day for 3 consecutive days. As shown in Figure 1B, mice receiving the antibiotic water, showed significantly higher *S. epidermidis* NRS122 CFU in the nose compared to mice receiving water alone, consistent with prior findings that clearance of endogenous flora facilitates colonization of nasally applied bacteria [15].

### Prophylactic application of *S. epidermidis* prevents MRSA colonization

**Figure 2:**
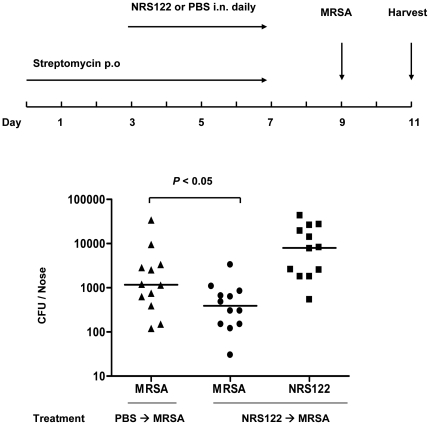
Pre-colonization of mouse nares with *S. epidermidis* NRS122 reduces colonization with MRSA. Top panel: Experimental design. Bottom panel: Comparison of MRSA colonization in mice given streptomycin water and pretreated with either PBS or *S. epidermidis* NRS122. Streptomycin-resistant strains of MRSA and NRS122 (5×10^8^ CFU) were applied at each inoculation. Bars in the graph represent the median CFU of each experimental group. Mean bacterial counts were 4715 CFU of MRSA in the control group, and 673 CFU of MRSA and 12953 of NRS122 in the *S. epidermidis* NRS122 pretreated group.

Using these optimized conditions, we investigated whether application of NRS122 could effectively reduce nasal colonization by MRSA. For the experiment, we pretreated mice with streptomycin, then inoculated one group intranasally with PBS, and the other group with streptomycin-resistant *S. epidermidis* NRS122. After two days, the mice were administered streptomycin-resistant MRSA by the intranasal route, and sacrificed after another 2 days. As shown in Figure 2, the mice that received streptomycin plus NRS122 showed reduced colonization with the MRSA compared to the mice that received streptomycin alone (Mean: 673 versus 4715; Median: 390 versus 1170). The number of competing NRS122 at the time of harvest was 12953.

## Discussion

The current study was undertaken to establish a murine model and to provide proof of principle that a probiotic strategy could prevent MRSA colonization in setting of close contact with MRSA colonizers such as nursing homes. It has been well documented that use of nasal mupirocin provides only short term eradication of MRSA in patients previously colonized with MRSA [2]. Within months, patients are colonized at the same rate as those who did not receive mupirocin treatment [2]. Mupirocin is active against a wide range of Gram-positive bacteria and many Gram-negative bacteria [16]. Therefore, application of the topical antibiotic could lead to eradication of the endogenous nasal flora, and without replacement flora, would permit MRSA to colonize. Alternatively, individuals who receive antibiotic treatment for any infection could become susceptible to MRSA colonization because of eradication of competing nasal flora.

Studies in the 1960's have shown that a less pathogenic *S. aureus* strain could be used to successfully outcompete the epidemic *S. aureus* strain [4]. Yet, the inherent danger associated with the application of a pathogen to displace another pathogen makes that an impractical approach [5], and led to the abandonment of that strategy. More recent and widespread use of probiotics for the prevention and treatment of a number of human gastrointestinal conditions have demonstrated that application of non-pathogens is relatively safe and likely more acceptable if applied for prevention of nasal MRSA colonization.

In the current study, we demonstrated that presence of an applied probiotic could prevent nasal colonization with MRSA. We showed that intranasal administration of a *S. epidermidis* strain reduced MRSA colonization two days later. From a translational viewpoint, this could be construed to be a preventative strategy for an acute MRSA outbreak. However it begs the greater question whether this could also represent a long term strategy that prevents MRSA colonization months later in high risk settings such as nursing homes. For that purpose, our data only support the concept that if there are sufficient competitor bacteria present at the time of MRSA exposure, the host will be less susceptible to nasal colonization with MRSA. However, the mouse model has its limitation for long term studies: Neither *S. epidermidis* nor MRSA colonize mice at high concentration or for an extended period of time, thereby limiting our ability to address that question. But even without additional animal data, existing published studies suggest that the strategy could work. For example, multiple studies have identified the antagonistic relationship of *S. aureus* versus *Corynebacterium* spp. or *S. epidermidis*: It has been shown that presence of *Corynebacterium* spp. or *S. epidermidis* often predicts the absence of the other species in human noses [8], [12]. In a longitudinal study of 166 premature infants in a neonatal intensive care unit, it has been shown that presence of *viridans* group streptococci in the first 2 weeks of life correlated with protection from MRSA colonization at the time of discharge from the hospital (9.5% versus 44.7%) [7].

Furthermore, in a study carried out at Nagano Children's Hospital, Japan, Uehara and coworkers enlisted the participation of 17 human healthcare volunteers who were persistently colonized with *S. aureus* [12]. They applied *Corynebacterium* spp. to the nares of these individuals and showed that inoculation of the competing bacteria led to *S. aureus* decolonization in 71% of the individuals [12]. The authors noted that *S. aureus* recolonization was not observed when the subjects were followed for 3–35 months suggesting that bacterial interference could lead to long-term decolonization. However, there were no control subjects to evaluate the rate of recolonization in subjects not given *corynebacterium* spp.

Among the key factors that are believed to play a role in inter-bacterial competition, binding to host receptors and secretion of bacteriocins are perceived to be important strategies used by competing microbes [1], [11]. Certain bacteria such as *H. influenzae* induce the recruitment of immune cells to drive the clearance of their niche competitor, *S. pneumoniae* [17]. It has been proposed that *S. aureus* and *S. epidermidis* limit cross-competition by secreting autoinducing (quorum sensing) peptides (AIPs) [18]. There are 4 alleles of AIPs corresponding to four distinct *agr* groups, and these peptides modulate the *agr* global regulator which in part regulates colonization. The cognate peptide from one *agr* group, upon secretion, activates *agr* expression in the same *agr* group but could inhibit *agr* expression in other staphylococcal groups. Therefore this could be a basis for suppression of colonization among staphylococcal species [18].

Our recently published study indicated that a subset of *S. epidermidis* secretes Esp, which inhibits *S. aureus* biofilm formation and destroys pre-existing *S. aureus* biofilms, and hinders *S. aureus* nasal colonization by novel interferential mechanisms [13]. In an assay for Esp, we determined that the interfering strain used in the present study produced Esp; however, the activity was strikingly weak (data not shown). Therefore, the interference observed in our model is likely due to another factor in addition to Esp. Future experiments will explore the relative contribution of various mechanisms towards bacterial interference using *S. epidermidis* and other probiotic strains. Such studies will provide interesting information for developing future therapies.

In summary, the emergent MRSA epidemic and the lack of a successful preventive strategy prompts the question of whether the timing may be ripe for re-evaluation of bacterial interference. The “probiotic” regimen, as determined by the bacteria to be included in the probiotic cocktail and the frequency of administration, requires further optimization, preferably in an animal model as established in this study. Because MRSA can colonize sites outside the nose, the efficiency of this approach towards preventing colonization elsewhere will also need to be evaluated. However, unlike antibiotics, which can become obsolete over time due to acquisition of resistance, bacterial interference may be more durable. In the absence of a vaccine, a probiotic applied intranasally and possibly outside the nose may offer a long-term solution not addressed by current MRSA preventative regimens.

## Materials and Methods

### Ethics Statement

All animal experiments were approved by the Cedars-Sinai Committee on the Use and Care of Animals and performed using accepted veterinary standards (IACUC protocol 2052).

### Bacterial strains, media, mice, and reagents

*S. epidermidis* NRS8 and NRS122 were obtained from the NARSA repository (www.narsa.net). *Corynebacterium* spp. strains 1 and 2 were skin isolates obtained from the Cedars-Sinai clinical laboratory. MRSA BD02-31 is a pulse-type USA500 strain (courtesy of Dr. Binh Diep). *S. epidermidis* and *S. aureus* strains were cultured at 37°C in either Todd-Hewitt broth (THB) or on Todd-Hewitt agar (THA) (Difco). Brain Heart Infusion (BHI) (Difco) broth and agar were used to grow *Corynebacterium* spp. When included, streptomycin sulfate salt (Sigma-Aldrich) was added to bacterial growth media at 500 µg/ml or to antibiotic water at 1000 µg/ml. CD1 mice were purchased from Charles River Laboratories, Wilmington, MA.

### Selection of streptomycin-resistant *S. epidermidis* and MRSA

*S. epidermidis* NRS122 and MRSA BD02-31 were grown overnight in THB, washed once in PBS (Dulbecco), and plated on THA with streptomycin (500 µg/ml). Streptomycin-resistant strains were isolated from the plates two days later. These strains were maintained and grown in streptomycin prior to *in vivo* studies.

### Murine nasal colonization studies

Bacteria used for nasal inoculation were cultured for 18–24 h in THB with or without streptomycin (500 µg/ml), and washed once in PBS. Mice were inoculated intranasally with 10 µl droplet of the inocula at the indicated concentrations. For bacterial enumeration, the mice were euthanized using isoflurane followed by cervical dislocation, and the nasal tissue was homogenized and vortexed for 5 min in PBS, and the homogenate was plated on THA with or without streptomycin after appropriate serial dilutions. Bacterial identification was based on antibiotic resistance patterns, colony morphology, and color as previously described [10]. Briefly, we have shown that mice (n = 5) administered PBS alone in the nose, harbor on average 1.9×10^6^ CFU per nose, but none of the endogenous bacteria grew on streptomycin (500 µg/ml) plates (Figure S1). Therefore, the inoculated streptomycin-resistant *S. epidermidis* NRS122 and MRSA BD02-31 could be clearly distinguished from the endogenous flora by growth on streptomycin plates. *S. epidermidis* NRS122 could be differentiated from MRSA BD02-31 on the basis color (white versus orange) on THA plates.

### Statistical analysis

Data were analyzed using Prism 4.03 (Graphpad Software, Inc.) and Excel (Microsoft). The results of the *in vivo* challenge studies were evaluated using the Mann-Whitney test. The Kruskal-Wallis test was used when three or more groups of data were compared. Unless otherwise indicated, a *p* value less than 0.05 was considered significant, and noted in the figures.

## Supporting Information

Figure S1 **Endogenous nasal bacteria from mice are susceptible to streptomycin.** CD1 mice (n = 5) were administered PBS intranasally daily for 3 days. On day 4, bacteria from the nares were plated on THA with or without streptomycin (500 µg/ml) plates.
(TIF)
