# Supplementary figures and images for: Intranasal Application of S. epidermidis Prevents Colonization by Methicillin-Resistant Staphylococcus aureus in Mice
Source: PLoS One. 2011 Oct 5;6(10):e25880. doi: 10.1371/journal.pone.0025880 (PMC3187813; doi:10.1371/journal.pone.0025880)

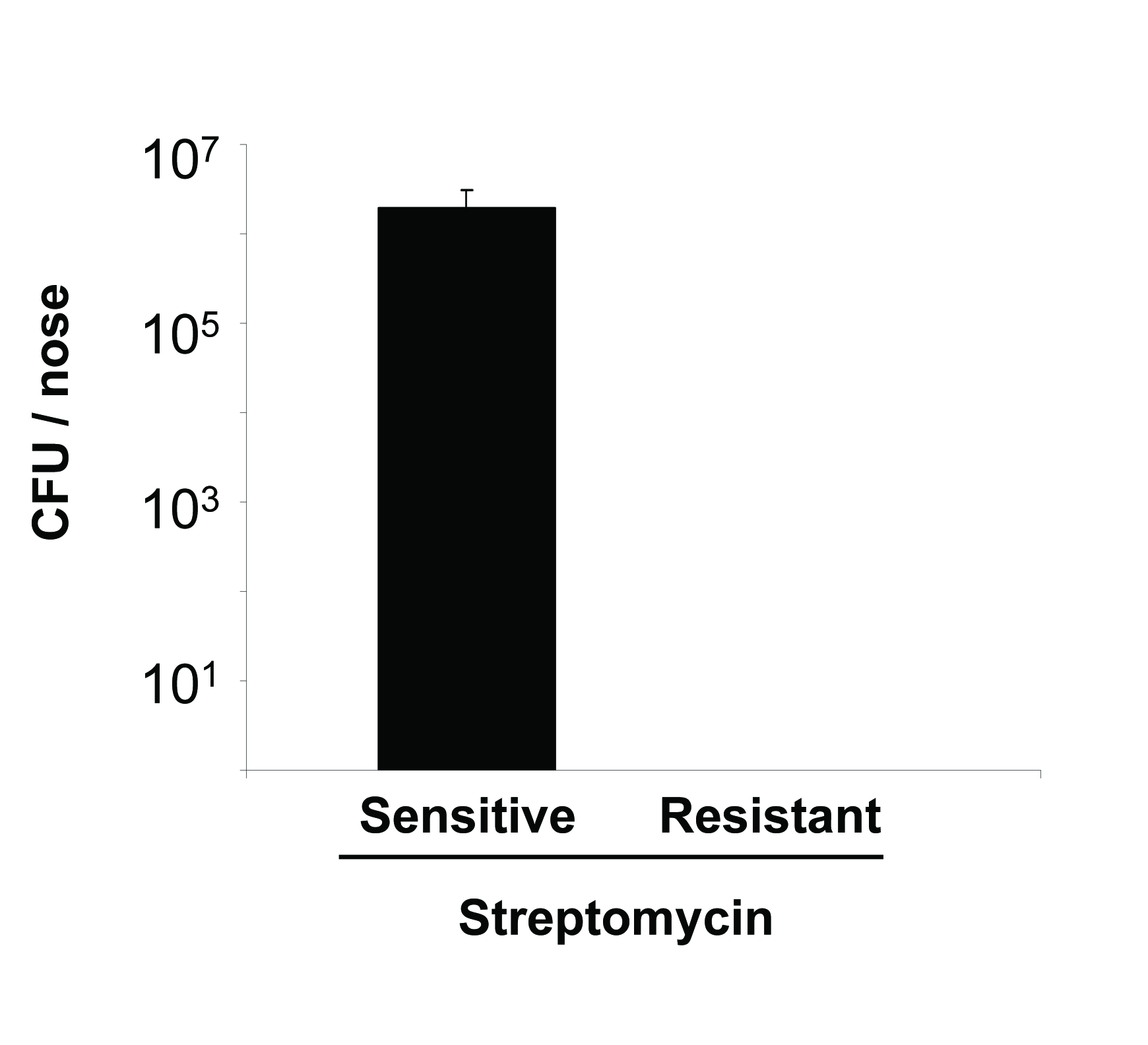

Supplement: Figure S1 — Endogenous nasal bacteria from mice are susceptible to streptomycin. CD1 mice (n = 5) were administered PBS intranasally daily for 3 days. On day 4, bacteria from the nares were plated on THA with or without streptomycin (500 µg/ml) plates. (TIF) [file pone.0025880.s001.tif]
